# Feasibility and acceptability of an online mental health intervention for pregnant women and their partners: a mixed method study with a pilot randomized control trial

**DOI:** 10.1186/s12884-023-06031-4

**Published:** 2023-10-18

**Authors:** Shannon M. Canfield, Kelli E. Canada, Abigail J. Rolbiecki, Gregory F. Petroski

**Affiliations:** 1 Department of Family and Community Medicine, University of Missouri, Columbia, MO USA; 2 School of Social Work, University of Missouri, Columbia, MO USA; 3 Department of Family Medicine and Division of Geriatrics, University of Colorado Anschutz Medical Campus, Aurora, CO USA; 4 Department of Biomedical Informatics, Biostatistics, and Medical Epidemiology, School of Medicine - University of Missouri, Columbia, MO, USA

**Keywords:** Perinatal mood and anxiety disorders, Pregnancy, Depression, Anxiety, Online intervention, Cognitive behavioral therapy, Couples, Social Support, Randomized control trial, Feasibility study

## Abstract

**Background:**

Untreated perinatal mood and anxiety disorders (PMAD) have short- and long-term health and social consequences; online cognitive behavioral therapy (CBT) interventions can reduce symptoms. Despite partner support being protective online interventions rarely target couples. This study builds on research on an existing CBT-based intervention, the *Mothers and Babies Online Course (*eMB), by testing its feasibility with prenatal couples.

**Methods:**

We conducted a pilot, randomized, controlled feasibility trial using a 1:1 parallel design. To be eligible, participant dyads were pregnant people (between 13–30 weeks gestation and with a score of 10 or greater on either the GAD-7 or PHQ-9 scale indicating elevated symptoms of anxiety or depression) and their cohabitating partners, living in Missouri, with access to the internet; both in the dyad consented to participate. Recruitment occurred via Facebook ads, flyers, and a snowball approach. The intervention group received eMB, and the control group received a list of community resources. We examined retention and adherence data extracted from eMB analytics and study databases. All participants were given depression and anxiety scales at baseline, 4 and 8 weeks to test preliminary efficacy; satisfaction and acceptability were measured at trial end (i.e., eight weeks) and via interview.

**Results:**

There were 441 people who responded to recruitment materials, 74 pregnant people were screened; 19 partners did not complete enrolment, and 25 dyads were ineligible. There were 15 dyads per group (*N* = 30) who enrolled; all completed the study. The survey response rate was 90% but partners required nearly twice the number of reminders. No participant completed all lessons. Mean depression and anxiety scores dropped over time for dyads in control (*M* = -1.99, -1.53) and intervention (*M* = -4.80, -1.99). Intervention pregnant people’s anxiety significantly decreased (*M* = -4.05; 95% CI [0.82, 7.27]) at time two compared to control. Twelve pregnant people and four partners participated in post-intervention interviews and suggested improvements for eMB.

**Conclusion:**

Online dyadic interventions can potentially reduce PMAD symptoms. However, to feasibly study eMB with couples, strategies to increase program adherence are necessary. Tailoring interventions to overtly include partners may be advantageous.

**Trial registration:**

ClinicalTrials.gov NCT05867680, 19/05/2023.

## Background & significance

Perinatal anxiety and depression are common, often co-morbid, and occur more frequently than outside pregnancy [1–3]. Untreated anxiety and depression during the prenatal period are associated with increased risks of pregnancy- and newborn-related complications, including birth complications, the likelihood of poor mental health postpartum, poor infant health outcomes and developmental delays, and problematic maternal-infant attachment [4–7].

The antenatal period is an optimal time for intervention [8]. Efficacious interventions include engaging pregnant people in cognitive-behavioral therapy and increasing the pregnant person's perception of social support —particularly partner support [8, 9]. A partner may or may not be the biological father and is a person providing behavioral as well as psychological support, in a committed couple, typically as part of a long-term relationship [10]. Despite the evidence that partners can buffer stressors and, more generally, that perceived social support is protective, there are few online perinatal interventions to reduce symptoms of anxiety and depression that involve partners [11, 12].

Online interventions are routinely used to manage mental health [13], increase knowledge and access to resources [14], and improve social connectivity [15]. Healthcare providers and allied health professionals — particularly primary care clinicians, behavioral health specialists, and medical social workers — have utilized internet-based services for decades [16–20, 19, 21]. Online interventions have shown promise for decreased perinatal anxiety, depression, and perception of stress, increased awareness about mental health risk factors and protective strategies, and improved coping self-efficacy [21–23]. The United States Preventative Services Task Force promotes research to increase access to these types of interventions [8]. Although perceived partner support is protective against poor mental health for pregnant people, partners' potential roles in online interventions is an understudied area of intervention innovation [22–24]. Expanding the number of effective and equitably accessible online interventions is essential, given structural and social barriers to care [25, 26].

Engaging the pregnant person and their partner as a dyad in perinatal studies complicates recruitment, retention, adherence, and longitudinally measuring change; key aspects of intervention feasibility [27, 28]. After gaining permission for use and obtaining access to a complete copy of the programming from the developers [29–31], this pilot study engaged pregnant couples experiencing elevated symptoms of maternal anxiety or depression with an existing online psychoeducation intervention, the *Mothers and Babies Online Course* (eMB). The study had three primary aims to assess overall feasibility; 1) explore the feasibility of delivering eMB to couples by assessing recruitment, retainment, and adherence, 2) examine eMB's preliminary efficacy for reducing PMAD symptoms, and 3) describe participants' satisfaction and perceptions about eMB acceptability.

## Methods

This concurrent mixed-method study used a prospective experimental research design with a randomized control trial followed by qualitative interviews. This trial is registered at ClinicalTrials.gov (NCT05867680, 19/05/2023) and conforms to CONSORT guidelines in the protocol and reporting for this study [32]. Dyads were randomly assigned to the control (PDF of perinatal community resource weblinks) or treatment (eMB) group using a 1:1 parallel assignment. Treatment group participants received the intervention during the study period, and control participants received access after the study. Intervention group study participants were invited to post-intervention interviews exploring their satisfaction and perceptions of program acceptability. Given the study aims, we hypothesized it would be feasible to deliver the program to pregnant couples and a significant reduction in anxiety and depression symptoms in favor of the intervention group.

### Setting and sample

The study is set in Missouri, a state with high rates of perinatal morbidity and mortality including poor mental health during pregnancy; access to services are a challenge for many although internet is generally available to most residents [33, 34]. Given the wide variation of policies, sociocultural norms, and access to resources between states, we limited the sample to those residing in Missouri. We used a multi-pronged recruitment strategy for the trial: 1) snowball (e.g., information shared from a list serve email and through social media), 2) flyers posted in perinatal clinics, and 3) Facebook ads. Recruitment materials targeted adults (i.e., minimum 18 years) identifying as pregnant and included information about the study and program, contact information, and eligibility criteria. To be eligible, pregnant people were between 13- and 30 weeks gestation, with elevated symptoms (a clinically significant score of 10 or more) of maternal a) anxiety or b) depression using validated measures — the Patient Health Questionaire-9 (PHQ-9) or the Generalized Anxiety Disorder-7 (GAD-7) [35, 36]; although partners were screened, there was no requirement for them having elevated symptoms. The score of 10 or more on either scale are routinely used in clinical decision-making as the point to consider pursuing treatment for a mood or anxiety disorder [35, 36]. The woman and her adult partner were required to cohabitate, report being in a relationship, and live in Missouri with internet access. If the pregnant person or her partner did not meet these criteria or could not provide consent, they were ineligible.

As part of the final trial survey, intervention group participants were invited to a post-study interview. The study investigator contacted interested participants to arrange a Zoom interview lasting up to one hour.

### Intervention: *Mothers and Babies Online Course* (eMB)

The previously developed eMB is an online, asynchronous, self-administered intervention modeled after the efficacious and in-person *Mothers and Babies Course* [29–31, 37]. The course promotes its use by those in key support roles (e.g., partners, family, friends) to the pregnant person to increase understanding of PMADs and therapeutic approaches to managing associated symptoms [30, 37]. eMB modules are delivered using a username and password through the Qualtrics platform. The eMB developers gave this study’s unaffiliated research team a complete copy of eMB using the Qualtrics platform for administration and provided technical assistance as needed to implement the intervention for the purpose of this study. The only modification to eMB course content was the addition of Missouri-specific perinatal and COVID-19 weblinks to the existing “resources” tab.

The eight-week course includes psychoeducational modules containing YouTube videos, vignettes, interactive quizzes, homework, guided meditations, and downloadable resources that teach strategies to promote a healthy outcome for mothers and their newborns. eMB recommends users complete one lesson per week and revisiting previously viewed content as needed. Users can visit the following lessons in any order: 1) purpose and overview, 2) thoughts and my mood, 3) fighting harmful and increasing helpful thoughts, 4) activities and my mood, 5) pleasant activities help make a healthy reality, 6) contact with others and my mood, 7) planning for the future and graduation, 8) relaxation exercises [30, 37]. This study is the first to test the feasibility or preliminary efficacy of eMB with couples.

### Study procedures

#### Recruitment and enrollment

Interested people contacted the study investigator by phone, email, or through a form auto-generated after clicking on the Facebook ad. Next, the investigator emailed study information and scheduled screening phone calls. On the call, the investigator discussed the study details with individuals of the couple independently — first, the pregnant person, then on a separate call with the partner whose contact information was supplied by the pregnant person. The investigator excluded couples if either did not meet the inclusion criteria, endorsed suicide risk, could not comprehend the consent document, or did not agree to participate. If both people were qualified and wanted to enroll, the (unblinded) study PI used computer randomization to order the group assignment to control or intervention.

Each eligible person in the couple received an instructional email indicating their group placement and information about the frequency of study surveys. Control couples received a description of when they would complete study assessments and a list of local resources. Participants used Qualtrics© to provide consent. Once they indicated consent, skip logic directed them to the Time 1 (baseline) survey [38]. The University of Missouri Institutional Review Board approved this study (# 2017228).

#### Data collection

Researchers used REDCap, a HIPAA-compliant tracking database, to store data on recruitment and retainment (i.e., Facebook ad responses, dates of personal emails or phone calls) and additional details on screening outcomes. Each participant was assigned an alternative identifier to protect their identity upon enrollment. We retained the number of inquiries and exclusion reasons for those not enrolled. The study investigator tracked survey administration and response data in the REDCap database.

The study investigator retained and integrated each enrollee's GAD-7 results with the Time 1 (baseline) survey data which collected depression data using the Edinburgh Depression Scale (EPDS; [39] and participant characteristics and demographics. After the Time 1 survey, individual participants received a Qualtrics© link to their individual and distinct email addresses measuring anxiety (GAD-7) and depression (EPDS) every four weeks of the eight-week study period (i.e., times 2 and 3). Researchers emailed up to two reminders, sent at 7-day intervals, to non-responders. Each survey took about 10–15 min, and all participants received emailed Amazon e-Gift card incentives, increasing incrementally, for completing surveys (i.e., USD 10, 20, and 25 for respective surveys).

Participants in the intervention group received email instructions on eMB access after the couple completed the Time 1 survey. The study investigator directed people to independently complete one module weekly, using their email addresses to log in, and when prompted via email invitation, to complete the 4- and 8-week surveys (i.e., times 2 and 3, respectively). Emails containing Time 2 and 3 survey links reminded intervention group participants to continue the eMB at a pace of about one module per week. Researchers tracked and recorded the survey responses and whether reminders were sent. Post-intervention, the study PI conducted voluntary, one-on-one interviews with individuals of the intervention group if they indicated the desire to do so in response to an invitation question on the time 3 survey. The PI sent an email to those who wanted to participate in the interview to arrange an interview time and then provided a link for the online interview; interviews were conducted and recorded via Zoom. For eMB program completion information, we downloaded login and lesson completion data from Qualtrics©.

Control group participants were surveyed at the same frequency and following the same data collection protocols as the intervention group participants. These participants received information about mental health and community resources via a PDF each time they completed a survey. Control group participants received access to eMB after completing the Time 3 survey. Information contained in the PDF was identical to information available to eMB participants in the *Resources* area of the program site.

### Measures

Participants answered standardized survey items for gender, age, race, ethnicity, household income, education, and intimate relationship status from the Behavioral Risk Factor Surveillance System survey [40].

For feasibility measures, we gathered administrative data at enrollment and with each instance of survey administration for response rates and downloaded Qualtrics data analytics to evaluate eMB usage. The research team used these data to calculate recruitment, retention and attrition rates, and participant intervention adherence [28]. The recruitment rate is the proportion of study inquiries and enrollments. Retention is the portion of survey completions and the reminders at each measurement time for each person. Attrition is the proportion of enrolled dyads and dropouts (i.e., both members failed to complete the final two surveys). Intervention adherence is the average number of participants completing one lesson per week, the proportion of fully or partially completed lessons, and the average total number of lessons visited. An additional descriptive usage measure was captured with a multiple-option survey item asking whether the participant used eMB alone, together with their counterparts, a combination of alone and together, or not at all.

To evaluate preliminary efficacy, we examined anxiety and depression symptom severity at each survey time using the Edinburgh Postnatal Depression Scale (EPDS) and the GAD-7. The PHQ-9 was used as a depression screener eligibility with a cutoff score of 10 or greater — consistent with moderate symptoms of major depression — required for eligibility. The PHQ-9 is a self-report, nine-item scale that measures depression severity with high internal reliability (α = 0.89). Response options are on a 4-point scale with scores ranging from zero to 27 with higher scores representing more severe depression symptoms [35]. All scales are self-report and administered via Qualtrics.

The EPDS is a ten-item self-reported questionnaire validated for use in pregnancy, acceptable for use with partners, [39, 41] and found to have good reliability (α = 0.87). The item responses are scored from zero to three to indicate symptom severity. A total score ranges from zero to 30 with a score of ten or more indicating moderate symptom severity.

The GAD**-7** has seven self-reported items assessing general anxiety levels and has good reliability (α = 0.89). Response options range from zero to three based on symptom severity. Total scores range from zero to 21 [31] with higher scores indicating more severe anxiety symptoms [36].

We used the Client Satisfaction Questionnaire (CSQ-8) for evaluating program satisfaction [42]. The CSQ-8 is a 4-point Likert scale measuring the following factors: perceptions of eMB quality, if course was the kind they wanted, met needs, help given, helps deal with problems, overall satisfaction, whether the person would repeat the intervention or recommend it to a friend. Responses range from strongly disagree to strongly agree and total scores range from eight to 32. Higher scores indicate greater satisfaction. The scale has high internal consistency (α = 0.9) and is used routinely in healthcare research (Attkisson & Greenfield, 1995). Satisfaction and program acceptability were also measured qualitatively using a semi-structured interview guide for data collection. Qualitative interviews, conducted by the PI (SC) using Zoom, included questions prompting participants to describe the helpfulness of materials, ease of use, areas for improvement, and whether and how the course improved coping behaviors. We audio recorded the interview and transcribed via Zoom.

### Analytic approach

We followed Whitehead et al. (2016) guidance on sample size to complete a pilot feasibility trial and determined a sample size of 30 dyads (i.e., 60 people) with 15 dyad per condition was necessary [43]. We used univariate analysis to calculate descriptive statistics describing the sample. We used the same approach for feasibility outcomes.

To test the study hypothesis of preliminary effectiveness, we used a factorial Analysis of Variance (ANOVA) with the dyadic role and survey time as repeated factors. We used an intention-to-treat analysis approach and a significance level of *α* = 0.05 or less. Repeated measures were recorded three times for each participant and each outcome of interest to evaluate the effect of the eMB course. Based on the minimum Akaike Information Criterion (AIC), researchers used an optimal residual covariance structure for each outcome [44]. When factor interactions were significant, we used post hoc comparisons. The study retained data when available and dropped cases missing at Time 2 or 3. Data missing in this study are considered missing at random and analyzed with maximum likelihood estimation [45]. Analyses were conducted using SPSS software [46].

To assess the degree of program satisfaction using the CSQ-8 we summed item responses and calculated the average score to quantitatively evaluate the degree of satisfaction among intervention group participants.

### Qualitative analysis

Interview data were analyzed using an inductive thematic approach to analysis using the steps outlined by Braun and Clarke [47]. All authors are PhD-trained researchers with extensive experience conducting qualitative analyses in health and social science research studies [48, 49]. The analysis occurred in stages: preparing transcripts after Zoom download, becoming familiar with the data and creating memos, developing and applying coding (i.e., defining and then applying labels to text segments), searching for patterns in the coding, defining, and naming themes from patterns, and write-up of results [50]. During coding, interview data were deconstructed into chunks by reading through transcripts line by line using; open coding was first used and then codes were grouped together based on thematic similarities. During the next round of coding, they were compared, contrasted, and synthesized to define overarching themes. The analysis was organized in Dedoose [51]. The study investigator completed each stage first, and a the second researcher (KC) conducted an analytic audit. Discrepancies were discussed and deliberated with the first and second author until agreement was reached.

## Results

**Fig. 1:**
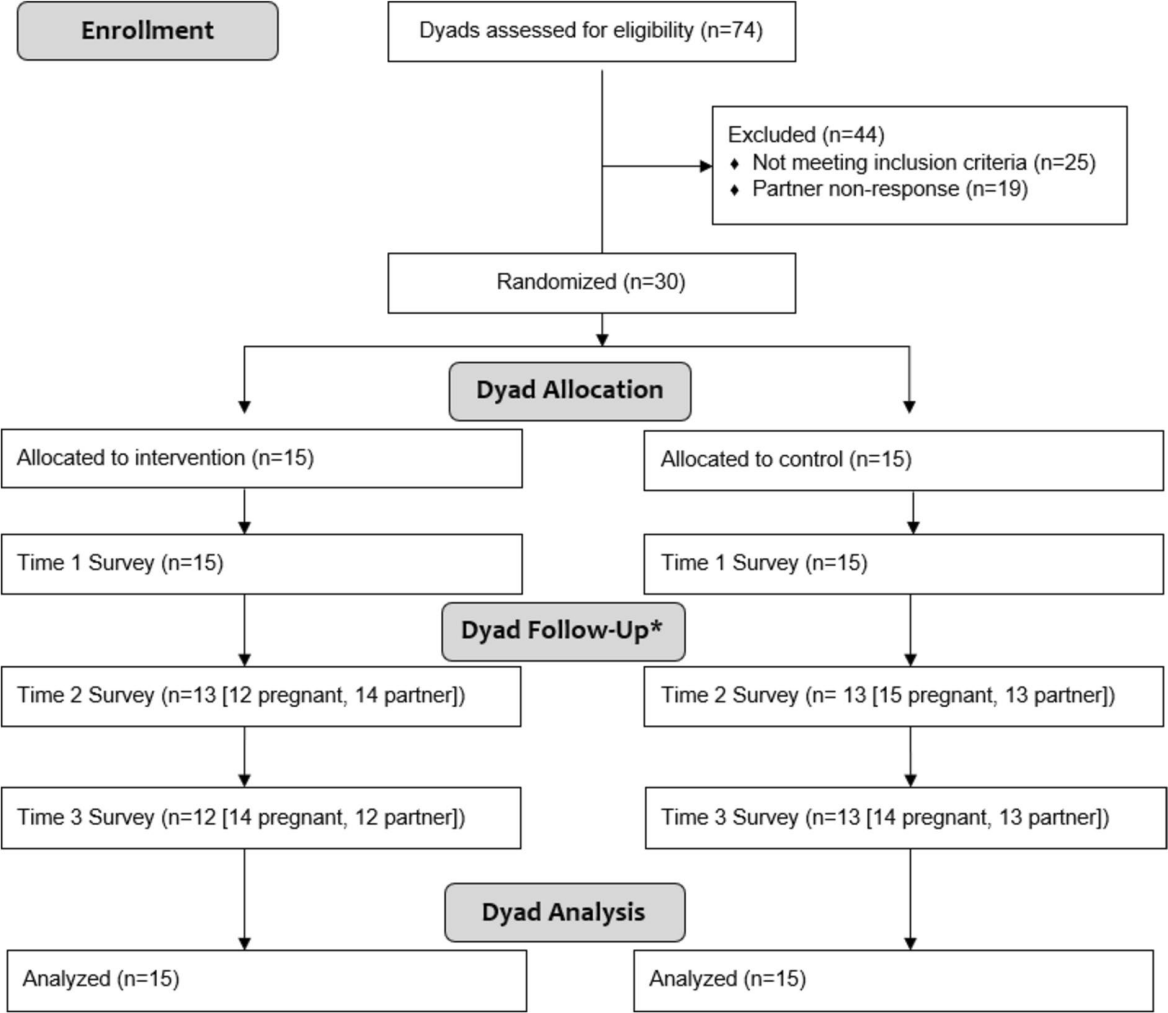
CONSORT flow diagram of dyad enrollment, follow-up and analysis

A total of 30 couples were enrolled in the study and randomly assigned to control (*n* = 15) or intervention. Figure 1 illustrates the study flow and participant responsiveness from recruitment through the end of the dyad's eight-week trial experience. The average age across groups was 31 years (*SD* = 7.8), with most identifying as married and non-Latinx White, some education beyond high school, and employed. Most participants reported an annual household income of USD 50,000 or greater; 23% of the control group and 13% of the intervention group reported USD 25,000 or less (see Table 1). We used the CONSORT checklist when writing our results, first in response to the study hypothesis and followed by the exploratory and descriptive results. There were no adverse events to report for this study.

**Table 1:** Study participant demographic characteristics

	**Control**	**eMB**
*N*	*30*	*30*
Age *M (range, SD)*	*31 (20–59, 7.1)*	*31 (18–51, 8.5)*
Race *%*		
White	*80*	*73*
Black/African American	*20*	*13*
More than one race	*0*	*13*
Ethnicity *%*		
Latino/a	*0*	*10*
Education *%*		
Some high school	*0*	*10*
High school or GED	*10*	*13*
Some college/technical	*30*	*37*
Undergraduate degree	*40*	*13*
Graduate degree	*20*	*27*
Employment Status *%*		
Employed	*80*	*80*
Homemaker	*7*	*7*
Self-employed	*3*	*3*
Student	*7*	*7*
Unable to work	*3*	*3*
Household Income *%*		
Less than 25,000	*23*	*13*
26- 35,000	*20*	*13*
36–50,000	*0*	*13*
51–75,000	*20*	*17*
75,000 or more	*37*	*43*
Relationship Description *%*		
Married	*80*	*80*
Never married	*20*	*17*
Separated	*0*	*3*

There were 12 pregnant people and four partners who participated in post-intervention interviews, and demographic characteristics were like that of the trial sample. Most interviewed participants identified as non-Hispanic White, with education beyond high school, employed, and a household income higher than USD 50,000.

### Feasibility outcomes

After removing duplicate inquiries, 441 interested pregnant people contacted the study investigator; most used the Facebook ad link (*n* = 414), and others called or emailed directly (*n* = 27). Of those who expressed interest, 74 coupled and pregnant people agreed to an eligibility screening call with the study investigator, and 44 met the inclusion criteria. Of the 44 coupled and pregnant, 30 had eligible partners and agreed to participate. Twenty-five dyads did not meet the eligibility requirements because the maternal anxiety or depression score was below the cut-off (*n* = 13), they did not live in MO (*n* = 4), or they were more than 30 weeks pregnant (*n* = 8). In 19 dyads, the partners did not follow up for the enrollment screening call. We ended recruitment after reaching the target enrollment number.  All 30 dyads remained in the study at Time 3, indicating zero study attrition.

Data were collected on a rolling basis from June to November 2020; enrollment ended after 30 couples enrolled, and the trial ended after the last couple completed the eight-week trial period. After the Time 1 survey, partners responded less often than pregnant people to surveys and required more reminders. In one control and two intervention cases, the partner did not respond to either the Time 2 or 3 surveys; in no case did this occur for pregnant people. In the intervention group, there were 26 pregnant people and 26 partner responses for Time 2 and 3 surveys; ten reminders went to partners and five to pregnant people. For the control group, there were 29 pregnant people and 25 partner responses with five and eight respective reminders for survey Times 2 and 3.

Of the intervention group participants who answered the usage question, 87% (*n* = 22) answered if they took the eMB alone or with their significant other. Half indicated having used eMB alone (*n* = 11), 9% (*n* = 2) reported together with their counterparts, 27% (*n* = 6) said a combination of alone and together, and 14% (*n* = 3 partners) indicated not engaging with eMB at all.

No participant followed the recommendation to complete one lesson per week. There were 83 discrete logins from six partners (*n* = 16) and 13 pregnant people (*n* = 67), with 24% (*n* = 21) of those being same-day logins. For any lesson visited, partners and pregnant people averaged 3.44 and 4.17 discrete logins, respectively. All lessons had at least partial completion by users, and all but Lesson 5 had at least one person fully complete the module; nine lessons were by a pregnant person and two by a partner. All users visited Lesson 11 except one pregnant person, and three pregnant people fully completed it. Otherwise, Lessons 2 and 4 were the most accessed. Lesson 8 had the least traffic, with three pregnant people visiting the material. Table 2 displays lesson completeness for individuals who logged into eMB.

**Table 2:** eMB lesson completeness

			Full (*n*) %		Partial (*n*) %		Total (*n*)
Lesson 1: Purpose & Overview	role*	p	0	0%	6	100%	6
		pp	3	23%	9	69%	12
	Total		3	16%	15	79%	18
Lesson 2: Thoughts & My Mood	role	p	0	0%	3	50%	3
		pp	2	15%	1	8%	3
	Total		2	10%	4	21%	6
Lesson 3: Fighting Harmful & Increasing Helpful Thoughts	role	p	0	0%	1	17%	1
		pp	1	8%	3	23%	4
	Total		1	5%	4	21%	5
Lesson 4: Activities & My Mood	role	p	1	17%	1	17%	2
		pp	1	8%	4	31%	5
	Total		2	10%	5	26%	7
Lesson 5: Pleasant Activities Help Make a Healthy Reality	role	p	0	0%	1	17%	1
		pp	0	0%	4	31%	4
	Total		0	0%	5	26%	5
Lesson 6: Contact with Others & My Mood	role	p	1	17%	0	0%	1
		pp	0	0%	4	31%	4
	Total		1	5%	4	21%	5
Lesson 7: Planning for the Future & Graduation	role	p	0	0%	0	0%	0
		pp	1	8%	1	8%	2
	Total		1	5%	1		2
Lesson 8: Relaxation Exercises	role	p	0	0%	0	0%	0
		pp	1	8%	2	15%	3
	Total		1	5%	2	10%	3

^*^p Partners, *pp* Pregnant People

### Overview of mean changes in outcomes

Both groups had reductions in anxiety and depression over time, indicating improvement in symptom severity from the Time 1 to 3 measurements. There was a more considerable reduction in both measures favoring the intervention group; however, the results were not found statistically significant with post hoc testing as described in the Preliminary efficacy outcomes section below. At Time 1, the intervention and control group mean anxiety scores were above the clinical cut-off of 10, indicating moderate anxiety symptoms (*M* = 11.07, *SD* = 5.55, *M* = 11.27, *SD* = 5.04). At Time 3, the intervention group scores decreased by 1.99 points, on average, indicating mild symptoms; the control group's mean decreased by 1.53 points which borders on moderate anxiety.

For depression, the mean scores at Time 1 was greater than 10, indicating moderate symptoms for participants in the intervention (*M* = 13.30, *SD* = 6.12) and control group (*M* = 12.03, *SD* = 5.40). At the end of the trial, the intervention group's mean score had dropped and indicated mild symptoms of depression (*M* = 8.50, *SD* = 4.07); the control group had a smaller reduction (*M* = 9.59, *SD* = 5.11). See Table 3 for an overview of mean scores and number of participants at each measurement time by group and role.

**Table 3:** Mean anxiety and depression by group, role and time

Scale	Group	Role	N	Mean (SD)	N	Mean (SD)	N	Mean (SD)
			Time 1		Time 2		Time 3	
EPDS	Control	Dyad	30	12.03 (5.40)	28	10.93 (4.66)	27	9.59 (5.11)
		Pregnant People	15	14.13 (4.42)	15	11.80 (3.88)	14	11.36 (4.40)
		Partners	15	9.93 (5.59)	13	9.92 (5.41)	13	7.69 (5.30)
	Intervention	Dyad	30	13.30 (6.12)	26	9.38 (3.89)	26	8.50 (4.07)
		Pregnant People	15	16.40 (4.81)	12	9.79 (4.54)	14	10.00 (3.58)
		Partners	15	10.20 (5.81)	14	8.92 (3.08)	12	6.75 (4.05)
GAD-7	Control	Dyad	30	11.27 (5.04)	28	10.32 (6.14)	27	9.74 (3.62)
		Pregnant People	15	13.07 (3.11)	15	11.93 (5.23)	14	9.93 (3.27)
		Partners	15	9.47 (6.01)	13	8.46 (6.78)	13	9.54 (4.10)
	Intervention	Dyad	30	11.07 (5.55)	26	8.35 (3.68)	26	9.08 (2.83)
		Pregnant People	15	14.80 (3.41)	12	7.79 (3.31)	14	9.00 (2.29)
		Partners	15	7.73 (4.72)	14	9.00 (4.11)	12	9.17 (3.46)

### Preliminary efficacy outcomes

We hypothesized the intervention group would experience a significant reduction in both anxiety and depression scores over time compared to the control group. Residuals were not grossly abnormal. However, there was a high-order interaction of role*group*time (*p* = 0.02) for the GAD-7 and role*time (*p* < 0.00) for the EPDS; therefore, we computed post hoc pairwise comparisons.

At Time 1, intervention and control group pregnant participants exceeded the clinical cut-off for moderate anxiety (*M* = 14.80 (*SD* = 3.41) and *M* = 13.07 (*SD* = 3.11). There was a significant decrease between groups for pregnant people's mean scores favoring the intervention group (*M* = -4.05, 95% CI [0.82, 7.27]). Women in the intervention group experienced significant point reductions between Times 1 to 2 (*M* = 6.92, 95% CI [4.36, 9.47]) and Times 1 to 3 (*M* = 5.78, 95% CI [3.83, 7.72]). Only between Times 1 to 3 did the pregnant people in the control group experience a significant decrease in anxiety scores (*M* = -3.18, 95% CI [1.23, 5.12]).

Between Time 1 to 2 and Time 1 to 3, pregnant people in the intervention group experienced a significant mean decrease in depression scores (*M* = 6.44, 95% CI 4.22, 8.66; *M* = 3.52, 95% CI 0.79, 6.25), as did partners for Time 1 to 3 (*M* = 3.52, 95% CI 0.79, 6.25; *M* = 3.52, 95% CI 0.79, 6.25). Intervention group partners' scores indicated mild symptoms and pregnant people experienced borderline moderate severity at the end of the trial. Women in the control group had significant reductions between Time 1 to 2 and Time 1 to 3 (*M* = *2.33, 95% CI 0.16, 4.51; M* = 2.70, 95% CI 0.46, 4.93) and remained above the cut-off for moderate symptoms at Time 3. In the control group, partners' depression scores significantly decreased between Times 2 to 3 only (*M* = 2.71, 95% CI 0.66, 4.76) and ended the trial with mild symptoms.

There were no significant between-or within-group differences for partners at any time in the study. For the control group, there were significantly lower mean scores for partners at Times 1 (*M* = -3.60, 95% CI [-7.29, 0.90]) and 2 (*M* = -3.51, 95% CI [-7.53, 0.50]) when compared to pregnant people. In the intervention group, mean scores for partners were significantly lower than pregnant people's at Time 1 (*M* = -7.47, 95% CI [-11.16, -3.78]). Table 4 illustrates changes over time by role and between groups.

**Table 4:** Time by role and group; Pairwise comparisons for anxiety and depression

	Time	Role^b^	(I) Group	(J) Group	Mean Difference (I-J)	Std. Error	df	Sig.^c^	95% Confidence Interval for Difference^a^	
									Lower Bound	Upper Bound
GAD-7	1	p	control	eMB	2.13	1.91	30	.27	-1.76	6.02
		pp	control	eMB	-1.73	1.15	30	.14	-4.08	0.61
	2	p	control	eMB	-0.004	2.07	28	.10	-4.24	4.23
		pp	control	eMB	4.05^c^	1.58	29	.02	0.82	7.24
	3	p	control	eMB	0.53	1.42	27	.71	-2.38	3.45
		pp	control	eMB	0.87	1.01	29	.40	-2.93	1.20
EPDS	1	p	control	eMB	2.13	1.91	30	.27	-1.76	6.02
		pp	control	eMB	-1.73	1.15	30	.14	-4.08	0.61
	2	p	control	eMB	-0.004	2.07	28	.10	-4.24	4.23
		pp	control	eMB	4.05^c^	1.58	29	.02	0.82	7.24
	3	p	control	eMB	0.53	1.42	27	.71	-2.38	3.45
		pp	control	eMB	0.87	1.01	29	.40	-2.93	1.20

Based on estimated marginal means

^a^Adjustment for multiple comparisons: Least Significant Difference (equivalent to no adjustments)

^b^p signifies partner, and pp signifies the pregnant person

^c^The mean difference is significant at the .05 level

### Participant satisfaction and perceptions of eMB acceptability

The average group score indicates excellent satisfaction (*M* = 26.00, *SD* = 2.58), as do scores by role (pregnant people *M* = 26.00, *SD* = 2.68 and partners *M* = 25.95, *SD* = 2.37). Overall, the group strongly agreed the course was easy to navigate (*M* = 3.42, *SD* = 0.55). Similarly, the by-role average indicates participants most strongly agreed that eMB was easy to navigate (pregnant people *M* = 3.42, *SD* = 0.59 and partners *M* = 3.43, *SD* = 0.49).

**Fig. 2:**
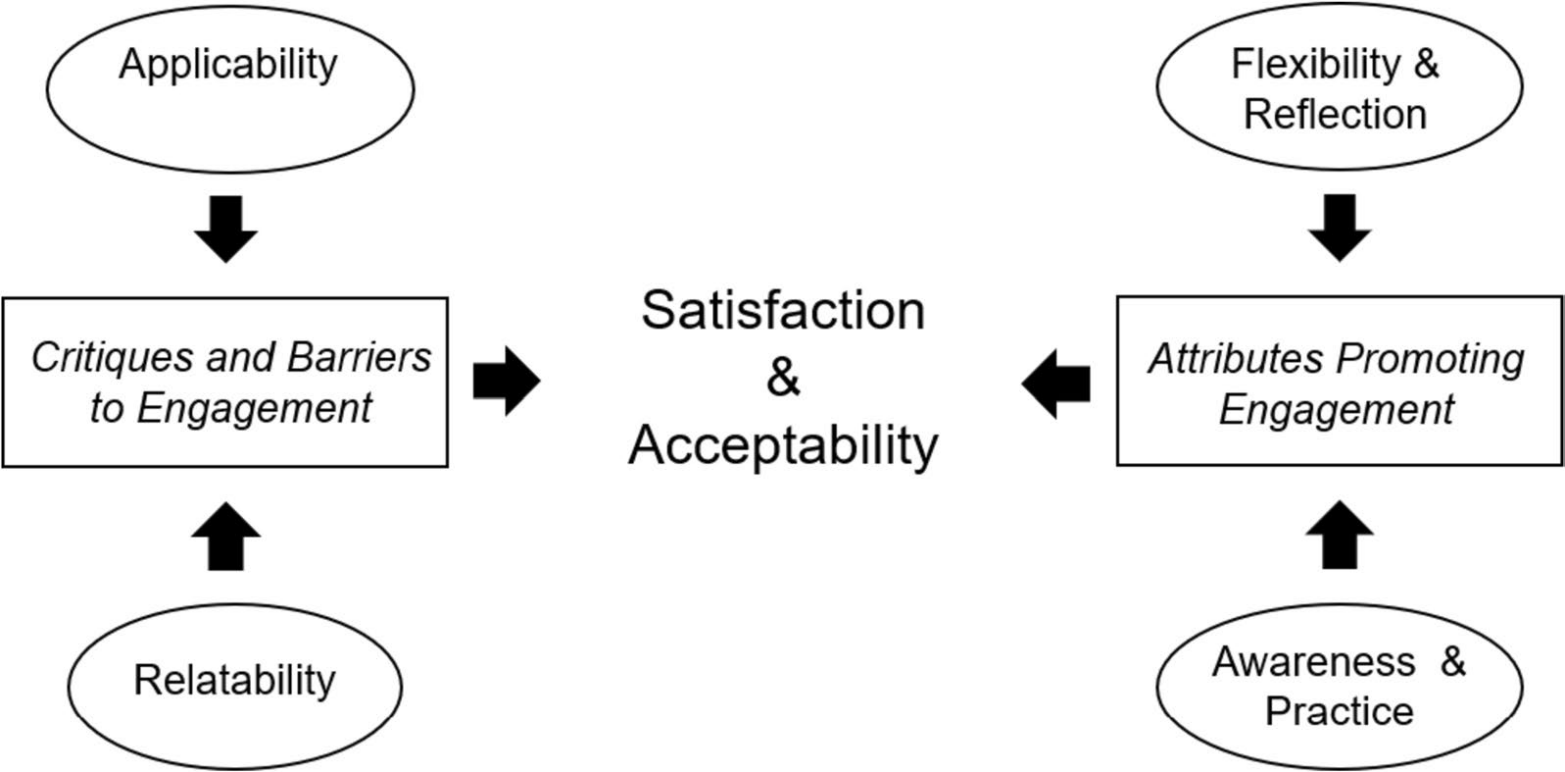
Primary and secondary themes of satisfaction and perceptions of acceptability when using eMB

Findings from interviews with participants generally support the conclusions from the satisfaction survey. Primary themes regarding participants' satisfaction with eMB when used with couples' center on the attributes of course engagement and critiques of the eMB experience; see Fig. 2.

### Attributes promoting engagement with eMB

This primary theme includes participants' descriptions of learning experiences, use of course materials, and their impressions of what they found as attributes promoting engagement with course content; *flexibility* and *increased awareness* define secondary themes. eMB flexibility refers to people's thoughts on how the course design and delivery are easy to use and how the course encourages different opportunities for reflection. Participants discussed the value of using the course independently as a convenience feature and because it allowed them to learn and process content at their own pace. To illustrate how participants perceived flexibility, this participant reported:

*"For me personally, I prefer doing it separately because it did give me the time to just focus on where I was [before]…talking to him about… what I was feeling… it let us really focus individually without really feeling like we had to cater to [the other], you know, because we weren't always on the same page…So, it really helped us know where we were individually and be able to talk it out." Pregnant Person ID 19*

Partners similarly perceived flexibility to be a key attribute of the intervention. For example,

*"It gives you these things to think about and then like you're thinking about like seven or eight things, and then you're like sitting there like watching TV, and you think of one, or she said something like triggers kind of one part of something like, 'Oh, hey, yeah, what about…' then you just go off on a, like a 20-minute conversation… We just kind of wanted to look at it alone and come back and see what each person kind of gleaned from it." Partner ID 4*

A couple might use the course together intentionally or not, and people described that flexibility as positive. It was planned and intentional for some, while others perceived the flexibility to be a matter of convenience. Participants also described a bystander effect where one person in the couple would be viewing eMB lessons, and the other would look over their shoulder and become interested enough to log on to the intervention or watch with their partner. The quote below illustrate couples' intentional and bystander experiences:

*" I can bring home [other] resources and be like, 'Hey, you should read this.' But usually, he's not going to. Like, it literally has to be like, I read something to him…It's annoying…He watched a couple videos with me…[other times] I opened my computer and start watching them, and so he'd lay there and watch them with me." Pregnant Person ID 62*

### eMB promotes increased awareness and to practice of coping strategies

The eMB focus on mental health awareness and learning coping strategies were described as necessary. For some, these learning opportunities served as helpful reminders while encouraging them to think and act differently in certain situations. Participants suggested eMB helped them identify existing and new sources of support in various community networks. One participant described identifying with the course content and examples and how that helped her shift her perspective about reaching out for support:

*" [referring to an eMB vignette] It was like a conversation between her and a friend, and it was actually quite realistic. Like, I think I've had been in a situation like that…when I'm upset and I'm going through things I do want to be alone, and I feel like that's the best way to handle it. But this offered a different alternative, and I thought that was great.*

*… I don't have great coping mechanisms. I've got depression and anxiety. Like, I'm struggling with that. So, to see them offer solutions to things that I had been doing technically wrong in the past, I thought that was really helpful because now, instead of saying, 'Just leave me alone, I need space.' I'm going to work through it myself.' I'm second-guessing that and say [to myself], 'Okay, well, maybe I should talk to someone about it, maybe I should talk to a friend and let them help me through it, too.' Yeah." Pregnant Person ID 70*

Participants noted specific exercises as helpful in developing concrete ways to incorporate changes relating to thoughts, actions, and behaviors associated with positive coping. For some, the content helped people think through past times that were distressing and have concrete ways to cope and others described the practice as preparation for upcoming transitions or stressors.

*"The, the course is the target about in really going through what are the thoughts that I've been experiencing and how to respond to those thoughts in a helpful way…How to address them, or I guess refocus myself on to something more positive…building confidence and know that you know, 'We had a bad experience with our first pregnancy, but there's hope for this one.' " Pregnant Person ID 19*

*"I like the fact that there is a variety and I liked, where would say, 'You know, give three examples of things that you know you want to work on.' or like having to write it out. I felt like really helps you, like, really think about how you're actually going to do this, you know, what are you actually going to do or what do you really want to work on." Pregnant Person ID 21*

The videos were helpful in presenting concept overviews and learning objectives. Participants described the videos as essential linkages to other content and implied that various teaching approaches worked well for their learning and relating to the content. One partner said, *"The videos are great, and the more interactive things online I found great as well." Partner ID 76.* This pregnant person shared a similar impression, and she noted appreciation for the engaging, fill-in-the-blank course activities:

*"I really like the exercises, because I don't know if it's just my personality, but I think you learn better when you're, you know, doing an exercise or an activity rather than just having someone talk at you for half an hour or whatever. So, I like that. And the videos are helpful with the little examples of, you know, 'This mom does this, and this mom did this.'" Pregnant Person ID 21*

### Critiques of the eMB experience

The satisfaction survey indicated that participants thought the course was generally satisfactory; however, interview results highlighted potential ways to improve the course. The second primary theme is *critiques of the eMB experience,* which includes ideas for improvement, ways eMB discouraged engagement or aspects that did not meet the user's expectation. Secondary themes include *relatability (*i.e., outdated, silly, or extreme) and participants' impressions of *applicability (*i.e., timely and relevant to one's needs).

### Relatability

Participants described how delivery affected the relatability of the program content. Poor video quality, outdated images, and simplistic, low-tech visualizations influenced users' engagement. Similarly, users perceived content as silly (e.g., comical-looking call-out conversation bubbles) or extreme vignettes and illustrations to contrast maladaptive versus adaptive coping behaviors. Most critiques were minor, and participants indicated the ability to look past aspects they disliked, but others were distracted and may not fully engage in the content. For example, this pregnant person noted stylistic qualities that interfered with her experience:

*"I'm watching, and I'm trying to just be positive about it, and I'm like, 'Oh, I could really use that,' but then it's an old course. Like you can tell that it's definitely dated. So, when it comes to someone my age, I'm [feeling] like, I don't know like, it's kind of boring because it's so old. Like I wouldn't sit there and watch a Western movie." Pregnant Person ID 62*

The vignettes included images and corresponding conversation callouts portraying adaptive vs. maladaptive behaviors of pregnant or postpartum people. Some participants described them as "cartoons" which may have been distracting or disengaging:

*"I think those [vignettes] were just a little silly, and they shouldn't have been silly because they're basic, and obviously I need this basic information...like I said, to refocus my thought process and stuff like that." Pregnant Person ID 81*

Participants suggested the course was not always relatable based on their own lived experiences. From this pregnant person's perspective, the eMB presented overly dramatized and unrelatable scenarios as part of lesson materials: *"So, those things [scenarios] were a little aggressive just for me because I never got that emotional. I'm a pretty even keel person, I feel like, most of the time anyway." Pregnant Person ID 2.*

### Applicability of eMB content

Applicability of eMB is about whether the content was informative and useable, thereby meeting the needs and expectations of participants. There were a range of perspectives. Some felt the course did not contain the expected or desired information about pregnancy or mental health needs in pregnancy. Some expected more generalized information about pregnancy (i.e., what to expect in pregnancy, at birth, and beyond). Others described the content as primarily applicable to first pregnancies. These quotes illustrate that the content did not satisfy all learner needs:

*"It was just really boring to me. I have other kids. So, this is my third. So, for me, it was kind of like everything they already tell you. But I do think like if you've never had a kid before and you're getting into that course, it's helpful." Pregnant Person ID 62*

*"I was just kind of hoping that it would be…like those books for idiots. You know, I mean like the ones that like spells it out, and very much in detail. It's like I don't know, like pregnancy for idiot father, you're saying, you know, something like that where like basically just assumes that you don't really have any idea what's going on, or what to do, already have it [figured out], and it just kind of spells it out." Partner ID 4*

Participants described dissatisfaction with not clearly defined partner-oriented materials or lessons on the partner role. Given that the course suggested being appropriate for people in support roles, including partners, people were surprised at no tailored content. This person's quote is representative of many participants:

*"Just maybe, like I said, the involving the partner, having a different version for the partner or relating it more to them…have a section on them about how they [pregnant people] might be perceiving their partners... if you're going to include them [partners] at least like I think it could be geared more towards them." Pregnant Person ID 40*

Overall, participants seemed satisfied with the eMB course. People noted ways the course could be improved for a better user experience by including better aesthetics and increasing content applicable to the partner or the couple collectively. Engagement with content was a primary theme inclusive of how the course inspired reflection about systems of support they had in their life, noticing and reframing thoughts from destructive to positive, thereby influencing actions (i.e., making positive choices).

## Discussion

PMADs are prevalent among pregnant people and their partners [12, 52]. This study is among the first to include the dyad in an online intervention to reduce symptoms of anxiety and depression for PMADs. The study successfully enrolled and retained a racially and ethnically diverse sample that was representative of the state, this promising result suggests the study’s approach informs methods with the potential to reach a large diverse sample, a commonly reported barrier in other studies [22]. This may be due to study procedures requiring frequent engagement between the researcher and participants or because of an agreement between the perinatal dyad. In-person prenatal service shortages and policy restrictions during the COVID-19 pandemic likely encouraged participation [17].

Despite pregnant people's eligibility in the study, many couples were not enrolled due to a non-responsive partner. Partners enrolled in the study responded less often to surveys than pregnant people, and only partners indicated never having engaged with eMB during the trial. Similarly, participants did not complete the lessons as directed and thus did not receive the total treatment dose. Given these results, future online studies may retain participants through a highly engaged intervention team and examine different approaches to engaging the perinatal unit to encourage treatment adherence and program completion [53].

Participants' satisfaction with eMB suggests a successful course translation from in-person programming to online delivery. The eMB course seemed to increase emotional awareness and connectivity and the potential to buffer risks associated with anxiety and depression. Despite high satisfaction scores, fewer partner participants engaged with the programming than pregnant people. This study's feasibility findings suggest the need for improving intervention adherence, a similar result as in other studies [54]. Additional measures to increase engagement may be advantageous and necessary for understanding if the amount of usage (i.e., dose) affects outcomes.

Symptom severity for anxiety and depression decreased in favor of the intervention group and statistically significantly reduced anxiety symptoms between groups for pregnant people at the study midpoint. Previous CBT-based online interventions for pregnant people found significant reductions in anxiety or depression at the study conclusion [23, 24]. The statistically significant decrease in anxiety for pregnant people suggests that prenatal psychoeducational programming to reduce symptoms of PMADs is helpful, a finding that supports previous research [23].

Given the relatively small change in partners' mood and anxiety scores compared to pregnant people in this study, future research warrants exploration of why pregnant people had significant decreases in anxiety symptoms. In contrast, the partner's changes were less dramatic. Evidence suggests relational factors [55] or a partner's poor mental health may be associated with perinatal pregnant people's outcomes [52]. Future studies with adequate power to examine dyadic pathways of individual couple members' mental health status as potential mediating or moderating factors on mental health outcomes are currently lacking in the literature [12, 56, 57].

More research on engaging couples in PMAD interventions is warranted, and findings from this study offer suggestions for improvement that may increase eMB engagement. Clinicians, behavioral health professionals, and researchers can partner with health departments, federally qualified health centers, or birth centers to examine using eMB [58]. The course may help overcome provider hesitancy to screen for PMADs due to the perceived lack of equitable and accessible treatment options [59]. From a prevention perspective, programs such as eMB have great potential to improve public health as a stand-alone resource or in combination with other treatments, education, and prevention tools [58, 59].

### Limitations

This study occurred during the COVID-19 pandemic and results are affected in numerous ways due to the historic event. The study recruitment was highly successful, and we meet our target enrollment within a few months; attrition was low. This study occurred in the early part of the pandemic and there was lacking perinatal education and mental health services available given most in-person encounters were stopped due to quarantine order therefore the recruitment and enrollment findings may not be suitable for estimating these outcomes in future studies. Further, the reliance on Facebook at the sole recruitment tool may have contributed to selection bias; future studies may consider multiple approaches for online recruitment.

Although the study had zero attrition, the parameters allowed for one or both people in the dyad to miss measurements therefore the analysis and interpretation of results are limited. The study design includes qualitative data collection and the findings from that inquiry provide valuable reflection on ways in which the pandemic shaped participants' perspectives. Understanding why some people did not adhere to study protocols is an important area of research for future study. As this study was a feasibility trial, the sample size was small as is appropriate in the examination of feasibility and for examining preliminary efficacy outcomes; the study is designed to inform future and adequately powered studies with generalizable results.

A limitation of this study was the lack of pre-intervention tailoring to adapt the content to the dyad; this likely contributed to the limited lesson completion. Future online studies aiming to decrease PMADs and engage the perinatal couple should use a design process that includes results from this study and engage dyads to gain additional insights for adaptation.Additionally, this study did not explore the potential effect of sociocultural factors and constructs such as social desirability; future research exploring these covariates would strengthen our understanding of dyadic interventions.

## Conclusion

The current study evaluated the feasibility and acceptability of eMB with couples and the preliminary effect on mental health outcomes, an essential contribution to the literature. There is a lack of PMAD intervention studies that include the couple, specifically those in the antenatal timeframe. Addressing PMADs within the perinatal couple can promote social support and has great potential to improve psychosocial outcomes for individuals and families. Programming such as eMB may be appropriate for couples, given adequate program engagement efforts and tailoring of information for partners. Further, online programing may decrease barriers to equitable and accessible resources for improving mental health. Family practitioners, OB-GYNs, and social workers serving perinatal people may find pregnant people benefit from online interventions promoting social support as part of a mental health treatment program. This study's findings have implications and can potentially affect policy, practice, and technologies that increase access to needed mental health care and promote prevention with a lifecycle perspective.

## Data Availability

The data that support the findings of this study are not openly available due to reasons of sensitivity and are available from the corresponding author upon reasonable request.
